# Analyzing Gait Pattern Associated With Neuropsychiatric Symptoms in Parkinson’s Disease by a Comprehensive Approach

**DOI:** 10.1109/JTEHM.2026.3700914

**Published:** 2026-06-05

**Authors:** Michela Russo, Carlo Ricciardi, Martina Mestizia, Federico Di Filippo, Noemi Pisani, Giuseppe de Biasi, Alfonso M. Ponsiglione, Paolo Barone, Marianna Amboni, Maria Romano

**Affiliations:** Department of Chemical, Material and Industrial Production EngineeringUniversity of Naples Federico II 9307 Naples 80125 Italy; Department of Electrical Engineering and Information TechnologyUniversity of Naples Federico II 9307 Naples 80125 Italy; Department of MedicineSurgery and Dentistry “Scuola Medica Salernitana,” University of Salerno Salerno 84131 Italy; Department of Advanced Medical and Surgical SciencesUniversity of Campania “Luigi Vanvitelli,” 80138 Naples Italy; IRCCS Synlab SDN Naples 80143 Italy

**Keywords:** Gait analysis, kinematics, kinetics, machine learning, neuropsychiatric symptoms, Parkinson’s disease

## Abstract

Objective: Parkinson’s disease (PD) is characterized by motor and non-motor symptoms. Among non-motor symptoms, neuropsychiatric symptoms (NPS), including depression, anxiety, cognitive decline and psychosis, are common and affect quality of life. The present study investigates gait patterns in PD patients with and without NPS, focusing on kinematic, kinetic, and spatio-temporal variables extracted from gait analysis. Notably, the assessments were carried out in a real-world outpatient clinical environment, which strengthens the translational value of the findings. Method: Overall, 104 PD patients were assessed using an optoelectronic system to record spatio-temporal parameters, kinematic and kinetic signals during a single task and two different dual tasks. First, a preliminary statistical analysis was carried out and then, several machine learning algorithms were applied to classify patients with and without NPS based on gait data. Results: Statistical analyses revealed that PD patients with NPS had slower gait, greater instability and reduced movement control. Decision Tree and Support Vector Machine classifiers showed promising results, particularly for kinematic and kinetic features (with an accuracy of 91.3% and 80.3%, respectively). The most recurrent features on which the classifiers were trained almost entirely matched the significance found by statistical analyses. Conclusion: The present findings further expand the relationship between motor and non-motor symptoms in PD highlighting that the relationship between gait and mental symptoms may extend beyond cognitive status. Clinical Impact—The study highlights the value of gait analysis as a promising, non-invasive surrogate biomarker for the early identification of a distinct clinical phenotype characterized by an increased burden of neuropsychiatric symptoms in Parkinson’s disease. The high accuracy achieved by machine learning models trained on gait features for distinguishing Parkinsonian patients with and without neuropsychiatric symptoms supports the development of assistive diagnostic tools that can enhance clinical decision-making, reduce subjectivity in patient assessments, and enable personalized monitoring of disease progression. Since the analyses were carried out in a real-world outpatient setting, the proposed approach naturally lends itself to integration within multidisciplinary clinical workflows, fostering synergistic collaboration among neurologists, physiotherapists and biomedical engineers, and contributing to a more personalized, precise, and proactive model of care.

## I. Introduction

Parkinson’s Disease (PD) is the second most common neurodegenerative disease whose diagnosis is primarily based on the identification of motor symptoms [1], [2]. However, the clinical presentation is heterogeneous and includes many non-motor symptoms that may occur at any stage of the disease course and may even precede motor symptoms [3], [4]. Among non-motor symptoms, neuropsychiatric symptoms (NPS), such as depression, anxiety, cognitive decline and psychosis, are frequent and increase disability and significantly affect quality of life [5], [6].

In this context, several studies have suggested that gait and postural control could serve as indicators of the health status of patients with PD; therefore, gait analysis has allowed the evaluation of gait patterns in PD [7], [8], [9]. For example, an increased range of motion (ROM) in the hip, knee, and ankle joints has been associated with camptocormia in PD [10]. Gait analysis is a 3D non-invasive method that provides quantitative details on locomotion. Various instruments, such as wearable and non-wearable sensors, allow a detailed analysis of gait characteristics, including spatio-temporal parameters, kinematic patterns, and kinetic measures [11]. For instance, Creaby and co-workers (2018) conducted a meta-analysis exploring the relationship between gait characteristics and the risk of falls in PD patients [7]. They found that alterations such as reduced gait speed, shorter step length, and postural instability were strongly correlated with an increased risk of falls. Additionally, Bouça-Machado et al. examined the gait parameters in PD patients, highlighting how changes in parameters such as step amplitude, gait speed, frequency, and step symmetry can be crucial indicators for monitoring disease progression and developing personalized treatments [8]. Numerous studies have demonstrated a strong relationship between gait and cognitive decline in PD populations [12], [13]. Specifically, Kim et al. found that gait patterns in PD patients with cognitive impairment were significantly more impaired than in those without cognitive deficits, under both single- and dual-task conditions [12]. Interestingly, Gaßner et al. highlighted that gait performance during dual-task conditions does not adequately reflect cognitive impairment, suggesting that more sophisticated methods are needed to assess cognitive decline in PD through gait analysis [13]. Additionally, gait has been suggested as a potential indicator for mild and subjective cognitive impairment, as well as neuropsychiatric symptoms [10], [14]. In our previous study, we examined the association between NPS and spatio-temporal gait parameters in a smaller cohort of PD patients, finding that the presence of such symptoms was associated with worse gait patterns, particularly during cognitive dual-task conditions [15].

Currently, the application of machine learning (ML) techniques for the automated recognition of gait patterns is expanding. In an increasing number of studies, ML algorithms have been used to identify specific motor patterns, classify different stages of various diseases, and predict critical events related to mobility impairments [16], [17], [18], [19], [20]. These advancements not only improve diagnostic accuracy but also contribute to the development of personalized treatment strategies and early intervention approaches, ultimately improving patient outcomes and quality of life [21].

In this study, we aim to further investigate the gait profile in PD patients with NPS (PD+NPS) compared to previous research, evaluating also kinematic, kinetic, and spatio-temporal patterns in single and dual task conditions and in a greater cohort of PD patients [15]. This represents a novel analysis in the literature, as kinematic and kinetic parameters have not yet been assessed in relation to the interaction between gait and NPS in PD. Importantly, the study was conducted in a real-world outpatient clinical setting, which enhances the validity and applicability of the findings. Indeed, two-way ANOVA statistical analysis was used to better evaluate the influence of the relationship between dual-task (motor and cognitive) and the presence or absence of NPS in such population. Subsequently, ML analysis was conducted to classify PD+NPS by better understanding the contribution of the kinematic and kinetic features.

## II. Materials and Methods

### A. Study Design & Population

In this study, 104 PD patients were consecutively enrolled at the Centre for Neurodegenerative Diseases at the University of Salerno (study authorized by the ethical committee Campania Sud with registry number “P.U. n.2_rpsc/2020” on 02/12/2020; informed was consent obtained by all patients). Each patient was diagnosed with PD according to the Movement Disorder Society (MDS) clinical diagnostic criteria for PD [22]. For each patient, motor and non-motor symptoms were evaluated using MDS-Unified Parkinson Disease Rating Scale (MDS-UPDRS) [23], and the progression of disease with Hoehn and Yahr Scale (H&Y) [24]. Traditionally, in clinical practice, the distinction between patients with and without NPS has typically been made on a clinical basis, using either general or condition-specific questionnaires. Specifically, MDS-UPDRS-Part I, evaluating mentation, behaviour and mood was used to characterize the dataset for this study. Therefore, based on the clinical opinion of the relevance of non-motor mental symptoms, the patients were classified as follows, according to an arbitrary cut-off:

• Sum of the first six elements (cognitive impairment, hallucinations and psychosis, depression, anxiety, apathy and dopamine dysregulation syndrome) of UPDRS part I *≥ 3* implied presence of clinically significant non-motor mental symptoms, resulting from at least half of the subitems showing any form of impairment (i.e., 3 subitems with a score > 0), or from two impaired subitems, at least one of which exhibiting moderate impairment (one subitem scored 1 and the second subitem scored 2), or even from a single severely impaired subitem (score = 3);
• Sum of the first six elements of UPDRS part I < 3 implied absences of clinically significant non-motor mental symptoms, because neither a severe impairment nor a moderate impairment within a multisymptomatic context can result from such a score.

In addition, cognitive abilities were screened with the Montreal Cognitive Assessment (MoCA) test. Then, they underwent gait analysis. Patients were recruited in a way that both the clinical assessment and the movement analysis were performed during the ON state, defined as the period of best therapeutic response according to the patient’s own report, usually in a time-window approximately ranging from 45 to 90 minutes after drug intake.

### B. Data Acquisition & Extraction

Gait was evaluated during the pharmacological ON phase using an optoelectronic system (SMART-DX 400, BTS-Bioengineering), in a real-world outpatient setting and during routine visits.

The system used for acquisition and elaboration was equipped with six infrared cameras, two force plates (60 cm x 40 cm) inserted in the center of walkway, two video cameras for walking recording, and dedicated software. The following steps, according to the Davis Heel protocol [25], were then performed in order:

1. Collection of anthropometric measurements (height, weight, leg length and pelvis diameter) of each patient.
2. Placement of 22 reflective markers on specific points of the patient’s body.
3. Standing phase on the force platform.
4. Walking phase on a straight path during three different tasks:
  a. GAIT (single task): the patient was asked to walk normally at self-selected speed.
  b. MOT (dual motor task): the patient was asked to walk while carrying a tray with 2 glasses filled with water.
  c. COG (dual cognitive task): the subject was asked to walk and simultaneously subtract the number 7 in series from 100.

**FIGURE 1.**
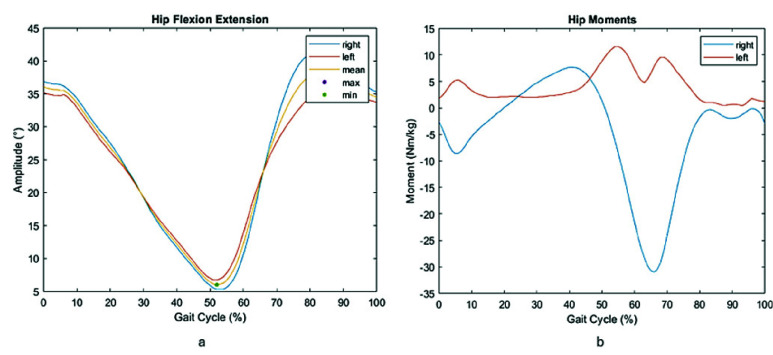
A): Hip flexion-extension pattern during the gaitcycle. The curves represent the average flexion-extension valuesof the right hip (blue line), left hip (orange line), and theoverall mean (yellow line). The points indicate the peak extension(purple dot) and peak flexion (green dot) of the mean curve.B): Hip moments during the gait cycle. The curves represent theaverage moment values of the right hip (red line) and left hip (greenline).

All patients completed four walking trials for each task; their average was used to calculate the parameters to improve the reliability of the data. Spatio-temporal parameters and sagittal plane kinematic and kinetic movements of the lower limbs (hip, knee and ankle) were calculated. The selected signals were used as input for a customized algorithm developed in MATLAB (version 24b), which calculated the maximum value, minimum value, and ROM for each parameter. This process generated a task-divided dataset which, together with the clinical-demographic dataset, was used to perform statistical and classification analyses using ML. Fig. 1 shows two examples of kinematic and kinetic signals.

### C. Statistical Analysis

First, a univariate statistical analysis for clinical and demographic variables was conducted using the IBM-SPSS software, after assessing the normal distribution of the data by means of the Kolmogorov Smirnov test. Normally distributed data were subjected to an independent samples t-test; otherwise, a Mann-Whitney test was employed. Subsequently, a two-way ANOVA was performed for spatio-temporal, kinematic, and kinetic parameters to identify differences between groups, assess the effect of the dual task and find interaction between symptoms-based groups and gait tasks. A significance level of *α =0.05* was adopted for all statistical analyses. For multiple post hoc comparisons, Bonferroni correction was applied.

### D. Supervised Analysis with Features Selection

After the statistical analysis, a supervised ML approach was implemented with MATLAB (v. R2024a). A classification analysis was conducted separately on spatio-temporal, kinematic, and kinetic parameters for GAIT, MOT, and COG, respectively.

First, parameter normalization and outlier removal were performed to improve the quality and consistency of the dataset. The following ML workflow included training, validation, and testing phases. 75% of the dataset was used for training and validation, while the remaining 25% was used for testing [26]. K-Fold cross-validation (K= 10) was applied to ensure the robustness of the predictive algorithms [27]. This approach allowed the model to be trained on different subsets of data and validated across multiple iterations, reducing bias and improving generalization.

Several ML algorithms widely reported in the literature for gait classification in patients with PD were employed [15], [16]. In the following study, 7 different classifiers were trained to determine which one provided the best results on gait data:

• Decision Tree (DT) in which data are recursively split into branches, with the leaves representing the class labels [28]).
• Support Vector Machine (SVM) that aims to find the best hyperplane that separates two classes of data, selecting the one with the greatest distance between the classes [29], [30]).
• K-nearest neighbors (KNN) that assigns each instance based on the closest training samples, using distance metrics like Euclidean or Manhattan distance [29].
• Linear Discriminant Analysis (LDA) that finds a linear combination of features to distinguish between two classes. It models class distributions using Gaussian distribution and is computationally efficient, even with high-dimensional data and limited training samples [29]).
• Naïve Bayes (NB) that works by computing the probability of each class and then selecting the class with the highest probability as the predicted class [29].
• Ensemble classifiers (Random Forest (RF) and Boosted Tree (BT)): RF is an ensemble of decision trees that improves accuracy and reduces overfitting by combining multiple independent models; BT is an ensemble model that builds trees sequentially, correcting the errors of previous ones to enhance accuracy, but with a high computational cost [29]).

For each proposed classifier, the performance was evaluated across multiple metrics to provide a comprehensive assessment of model: precision (Pr), sensitivity (Se), specificity (Sp), accuracy (Ac), and area under the curve receiver operating characteristic (AUC-ROC) [31]. Pr is a measure of the correctly predicted positive models out of the total predicted models in a positive class; Se and Sp represent the ability to correctly detect subjects belonging and not belonging to the group under examination, respectively; Ac is the ratio of correct predictions to the total number of records, and finally, AUC-ROC is a qualitative indicator for binary classification that ranges from 0 to 1 [32].

Moreover, to reduce the dimensionality of the data and find the best subset of spatio-temporal, kinematic, and kinetic parameters across the three gait tasks, a subsequent investigation was carried out using the feature selection method [33]. Specifically, the Chi-Square algorithm was implemented within the cross-validation process.

This feature selection algorithm uses the Chi-Square statistical test to measure the significance of each feature. It analyses the independence of each variable with respect to the target variable. If a variable is independent of the response, it is not used in the model; if there is a strong dependency, the feature is selected for the model [34].

**FIGURE 2.**
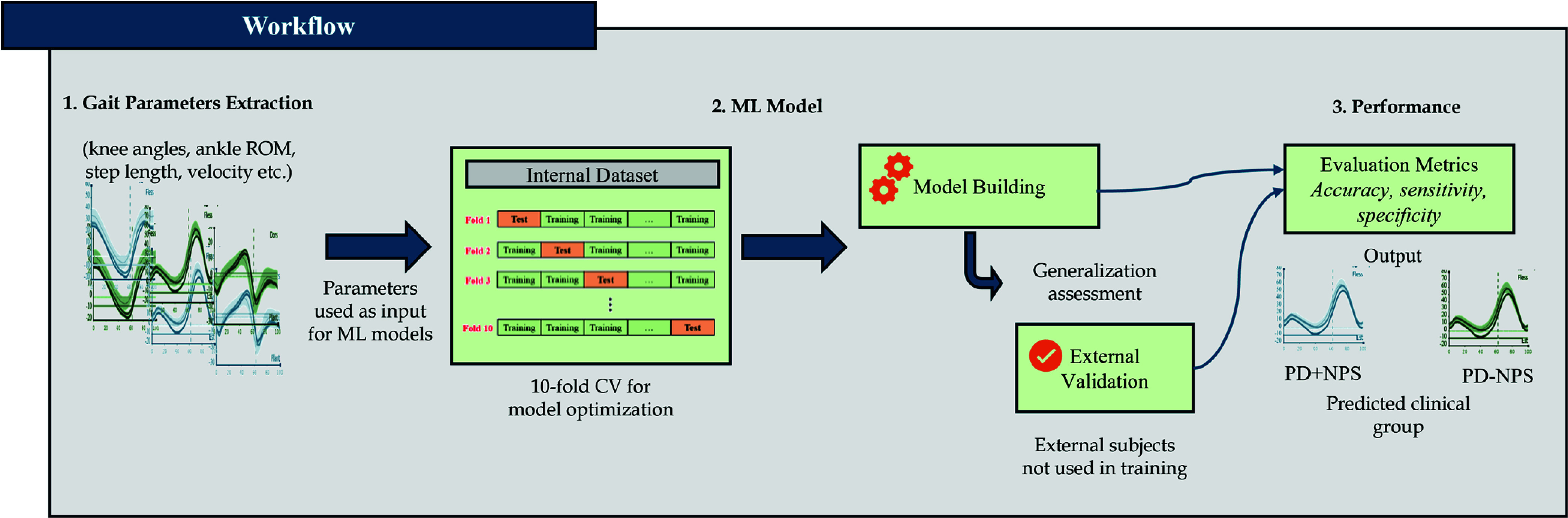
Workflow of the study: extraction of gait parameters, machine-learning model training with internalcross-validation, external validation, and final performanceevaluation.

Fig. 2 represents the overall workflow of the research.

## III. Results

### A. Demographical and Clinical Data Analysis

Among the 104 PD patients, 43 were classified as PD+NPS, while the others were PD patients without NPS (PD-NPS). As shown in Table 1, the two groups resulted matched for age, gender, disease duration, levodopa-equivalent daily dose (LEDD) and MoCA score. No significant difference was found in MDS-UPDRS Part III and IV score. Conversely, PD+NPS patients showed an increase in H&Y (p = 0.013), MDS-UPDRS-Part I (p< 0.001) and MDS-UPDRS-Part II (p< 0.001) scores compared to PD-NPS patients.

**TABLE 1:** Comparison of demographic and clinical characteristics between PD+NPS and PD-NPS by univariate statistical analysis (Mean ± standard deviation). Significant P-Values are highlighted in bold. Significance level at 0.05.

Clinical Demographic Variables	PD+NPS (n= 43)	PD-NPS (n= 61)	p-value
Age (years)	*64.02 ± 9.42*	*64.47 ± 8.34*	0.805
Gender	M= 25 F= 18	M= 44 F= 17	0.137
Disease duration (years)	*4.17 ± 2.16*	*4.69 ± 2.35*	0.193
LEDD (mg)	*525.15 ± 441.24*	*618.21 ± 726.49*	0.399
MoCA	*23.15 ± 3.51*	*23.18 ± 3.76*	0.898
H&Y score	*1.99 ± 0.32*	*1.81 ± 0.37*	0.013
MDS-UPDRS I score	*12.74 ± 6.25*	*4.93 ± 2.93*	< 0.001
MDS-UPDRS II score	*10.56 ± 6.55*	*6.62 ± 4.24*	0.001
MDS-UPDRS III score	*24.98 ± 9.59*	*22.08 ± 8.11*	0.166
MDS-UPDRS IV score	*2.02 ± 4.06*	*1.48 ± 2.46*	0.794
MDS-UPDRS total score	*50.30 ± 19.28*	*34.54 ± 13.49*	< 0.001

PD+NPS: Parkinson’s disease patients with neuropsychiatric symptoms; PD-NPS: Parkinson’s disease patients without neuropsychiatric symptoms; LEDD: Levodopa Daily Dose; MDS-UPDRS: Movement Disorder Society Unified Parkinson Disease Rating Scale; MoCA: Montreal Cognitive Assessment.

### B. Gait Analysis Data

The two-way ANOVA statistical analysis was carried out on spatio-temporal, kinematic and kinetic parameters, separately. The post-hoc tests were conducted for pairwise comparisons on gait tasks. As shown in Table 2, most spatio-temporal variables resulted significantly different between the two groups: stance duration (p= 0.017), stance phase (p= 0.006), swing phase (p< 0.001), single support phase (p= 0.003), double support phase (p= 0.017), velocity (p< 0.001), cadence (p= 0.050), cycle length (p< 0.001) and cycle length (% height) (p= 0.021). When assessing the influence of the task (GAIT, MOT, COG), the significant variables were cycle duration, stance duration, swing duration and variability, stance phase, single and double support phase, velocity, cadence, cycle length, and step length (all with p< 0.001), and swing phase (p= 0.016). Meanwhile, from the analysis of the interaction between group and task, the only significant variable was step length (p= 0.018).

**TABLE 2:** Comparison of Spatio-Temporal Parameters Between PD+NPS and PD-NPS by Two-Way ANOVA Statistical Analysis of Gait. MOT and COG Task (Mean ± Standard Deviation). Significant p-Values are Highlighted in Bold. Significance Level at 0.05

Spatio-temporal Parameters (u.m.)	Group	Task			Two-way Anova			
		GAIT	MOT	COG	Group	Task		Group * Task
							Post - hoc	
Cycle duration (s)	PD+NPS PD-NPS	*1.13 ± 0.12* *1.11 ± 0.12*	*1.11 ± 0.13* *1.10 ± 0.12*	*1.22 ± 0.20* *1.16 ± 0.14*	0.068	< 0.001	1-*3=0.002* *2-3< 0.001*	0.444
Stance duration (s)	PD+NPS PD-NPS	*0.69 ± 0.92* *0.67 ± 0.08*	*0.68 ± 0.10* *0.67 ± 0.08*	*0.77 ± 0.15* *0.73 ± 0.12*	0.017	< 0.001	1-*3< 0.001* *2-3< 0.001*	0.441
Swing duration (s)	PD+NPS PD-NPS	*0.44 ± 0.04* *0.44 ± 0.04*	*0.45 ± 0.16* *0.43 ± 0.05*	*0.36 ± 0.12* *0.33 ± 0.13*	0.099	< 0.001	1-*3< 0.001* *2-3< 0.001*	0.401
Swing variability	PD+NPS PD-NPS	*0.04 ± 0.06* *0.03 ± 0.02*	*0.04 ± 0.07* *0.03 ± 0.02*	*0.09 ± 0.06* *0.09 ± 0.06*	0.256	< 0.001	1-*3< 0.001~2*-*3< 0.001*	0.408
Stance phase (%)	PD+NPS PD-NPS	*60.84 ± 2.52* *60.39 ± 1.65*	*61.27 ± 2.22* *60.63 ± 1.91*	*62.73 ± 2.57* *61.76 ± 2.24*	0.006	< 0.001	1-*3< 0.001~2*-*3< 0.001*	0.693
Swing phase (%)	PD+NPS PD-NPS	*38.78 ± 1.96* *39.61 ± 1.69*	*38.72 ± 2.22* *39.42 ± 1.85*	*37.40 ± 2.58* *38.97 ± 4.43*	< 0.001	0.016	1-*3=0.034*	0.460
Single support phase (%)	PD+NPS PD-NPS	*38.63 ± 2.61* *39.62 ± 1.69*	*38.73 ± 2.22* *39.45 ± 1.92*	*37.50 ± 2.66* *38.30 ± 3.23*	0.003	< 0.001	1-*3< 0.001~2*-*3< 0.001*	0.921
Double support phase (%)	PD+NPS PD-NPS	*10.95 ± 2.00* *10.60 ± 2.69*	*11.88 ± 2.71* *11.28 ± 2.84*	*13.60 ± 3.74* *12.23 ± 2.39*	0.017	< 0.001	1-*3< 0.001~2*-*3< 0.001*	0.411
Velocity (m/s)	PD+NPS PD-NPS	*0.95 ± 0.18* *1.05 ± 0.16*	*0.93 ± 0.20* *1.02 ± 0.16*	*0.82 ± 0.21* *0.90 ± 0.17*	< 0.001	< 0.001	1-*3< 0.001~2*-*3< 0.001*	0.915
Velocity (% height/s)	PD+NPS PD-NPS	*58.02 ± 11.87* *61.97 ± 8.83*	*57.31 ± 12.01* *60.39 ± 9.23*	*50.68 ± 12.82* *53.52 ± 9.80*	0.007	< 0.001	1-*3< 0.001~2*-*3< 0.001*	0.928
Cadence (steps/min)	PD+NPS PD-NPS	*106.52 ± 12.09* *109.28 ± 11.43*	*109.31 ± 10.99* *110.83 ± 12.22*	*100.89± 14.10* *104.73± 12.78*	0.050	< 0.001	1-*3=0.013* *2-3< 0.001*	0.764
Cycle length (m)	PD+NPS PD-NPS	*1.06 ± 0.15* *1.15 ± 0.14*	*1.02 ± 0.17* *1.11 ± 0.15*	*0.97 ± 0.17* *1.04 ± 0.17*	< 0.001	< 0.001	1-*3< 0.001* *2-3=0.017*	0.774
Cycle length (% height)	PD+NPS PD-NPS	*64.82 ± 10.15* *68.11± 7.65*	*62.65 ± 10.80* *65.54 ± 8.45*	*59.85 ± 11.52* *61.45 ± 9.60*	0.021	< 0.001	1-*3< 0.001* *2-3=0.021*	0.782
Step length (m)	PD+NPS PD-NPS	*0.51 ± 0.09* *0.54 ± 0.12*	*0.49 ± 0.10* *0.53 ± 0.11*	*0.42 ± 0.12* *0.38 ± 0.14*	0.548	< 0.001	1-*3< 0.001* *2-3< 0.001*	0.018
Step length variability	PD+NPS PD-NPS	*0.14 ± 0.37* *0.19 ± 0.40*	*0.15 ± 0.46* *0.12 ± 0.28*	*0.21 ± 0.27* *0.15 ± 0.16*	0.741	0.596	-	0.496
Step width (m)	PD+NPS PD-NPS	*0.08 ± 0.03* *0.10 ± 0.05*	*0.09 ± 0.04* *0.10 ± 0.06*	*0.21 ± 0.27* *0.10 ± 0.05*	0.809	0.118	-	0.074

PD+NPS: Parkinson’s disease patients with neuropsychiatric symptoms; PD-NPS: Parkinson’s disease patients without neuropsychiatric symptoms GAIT: Single Task; mot: Motor dual-task; COG: Cognitive Dual-Task.

Overall, patients with PD+NPS vs PD-NPS showed: 1) reduced speed, as indicated by increased cycle duration and reduced velocity and cadence; 2) more instability, as revealed by increased stance phase, double support phase and swing variability; 3) less efficient gait, as suggested by reduced cycle length and decreased percentage of both single support and swing phase, indicating a reduced ability to transfer smoothly the body weight.

The post-hoc results confirmed the significance reported by the assessment of the influence of the tasks. In particular, the significant variables differed in the gait-cog (1-3) and mot-cog (2-3) tasks, but never in the gait-mot (1-2) tasks.

Table 3 shows the results of the statistical analysis conducted on kinematic parameters.

**TABLE 3:** Comparison of Kinematic Parameters Between PD+NPS and PD-NPS by Two-Way ANOVA Statistical Analysis of Gait. MOT and COG Task (Mean ± Standard Deviation). Significant p-Values are Highlighted in Bold. Significance Level at 0.05

Joint	Kinematic parameters (°)	Group	Task			Two-way Anova			
			GAIT	MOT	COG	Group	Task		Group * Task
								Post - hoc	
Hip	Flexion	PD+NPS PD-NPS	*32.34 ± 4.56* *31.46 ± 6.32*	*30.40 ± 7.18* *26.96 ± 6.64*	*32.07 ± 7.89* *30.66 ± 8.67*	0.873	0.929	-	0.233
	Extension	PD+NPS PD-NPS	-*4.65 ± 7.11* -*10.36 ± 8.07*	-*2.96 ± 6.91* -*6.40 ± 7.80*	-*7.59 ± 5.63* -*5.85 ± 7.07*	0.124	0.111		0.023
	ROM rotation	PD+NPS PD-NPS	*37.41± 5.99* *41.35 ± 4.81*	*33.36 ± 8.60* *32.66 ± 9.11*	*39.66 ± 8.48* *35.86 ± 10.05*	0.104	0.027	1-*2=0.001*	0.109
Knee	Flexion	PD+NPS PD-NPS	*56.35 ± 4.77* *58.83 ± 4.48*	*55.08 ± 10.64* *55.15 ± 8.90*	*52.76 ± 6.27* *54.96 ± 5.00*	0.232	0.006	2-*3=0.010*	0.452
	Extension	PD+NPS PD-NPS	-*0.74 ± 5.51* *2.55 ± 6.35*	*2.21 ± 4.69* *2.82 ± 6.81*	*1.19 ± 4.21* *1.35 ± 5.60*	0.010	0.082	-	0.975
	ROM rotation	PD+NPS PD-NPS	*57.57 ± 5.75* *54.28 ± 5.09*	*52.87 ± 9.41* *54.95 ± 17.82*	*51.57 ± 7.15* *53.61 ± 5.46*	0.683	0.078	-	0.536
Ankle	Flexion	PD+NPS PD-NPS	*12.22 ± 5.74* *11.89 ± 3.18*	*12.74 ± 3.90* *13.14 ± 2.91*	*12.05 ± 4.64* *11.64 ± 3.41*	0.875	0.091	-	0.975
	Extension	PD+NPS PD-NPS	-*19.19 ± 8.99* -*18.66 ± 7.60*	-*13.29 ± 6.36* -*13.62 ± 6.30*	-*13.79 ± 4.62* -*13.68 ± 5.83*	0.062	< 0.001	1-*3< 0.001* *1-2< 0.001*	0.385
	ROM rotation	PD+NPS PD-NPS	*31.41 ± 9.51* *30.55 ± 7.54*	*26.02 ± 5.94* *26.77 ± 5.70*	*25.84 ± 4.20* *25.32 ± 5.49*	0.098	< 0.001	1-*3< 0.001* *1-2< 0.001*	0.448

PD+NPS: Parkinson’s disease patients with neuropsychiatric symptoms; PD-NPS: Parkinson’s disease patients without neuropsychiatric symptoms GAIT: Single Task; mot: Motor dual-task; COG: Cognitive Dual-Task; ROM: Range of Motion.

The two-way ANOVA analysis conducted with reference to the group showed significant difference on knee extension (p= 0.010). Regarding the influence of the task, the significant variables were knee flexion (p= 0.006), hip ROM rotation (p= 0.027), and ankle extension and ROM rotation (both with p< 0.001). Meanwhile, regarding the interaction between group and task, only hip extension (p= 0.023) resulted significant. This means that the effect of the group on hip extension varies depending on the task. The post-hoc results confirmed the significance reported by the influence of the task highlighting that the variables mainly differed in the gait-cog (1-3) and mot-cog (2-3) tasks. Table 4 shows the results of the statistical analysis conducted on kinetic parameters.

**TABLE 4:** Comparison of Kinetic Parameters Between PD+NPS and PD-NPS by Two-Way Anova Statistical Analysis of Gait. MOT and COG Task (Mean ± Standard Deviation). Significant p-Values are Highlighted in Bold. Significance Level at 0.05

Joint	Kinetic parameters (u.m.)	Group	Task			Two-way Anova			
			GAIT	MOT	COG	Group	Task		Group * Task
								Post-hoc	
Hip	Extension moment in stance phase (N* m/Kg)	PD+NPS PD-NPS	*0.57 ± 0.19* *0.65 ± 0.18*	*0.66 ± 0.12* *0.66 ± 0.25*	*0.47 ± 0.17* *0.59 ± 0.16*	0.071	0.003	1-*3=0.017* *2-3< 0.001*	0.688
	Flexion moment in stance phase (N* m/Kg)	PD+NPS PD-NPS	-*0.61 ± 0.18* -*0.63 ± 0.34*	-*0.65 ± 0.23* -*0.62 ± 0.28*	-*0.53 ± 0.43* -*0.65 ± 0.27*	0.020	0.574	-	0.515
	Power generated in stance phase (W/Kg)	PD+NPS PD-NPS	*0.80 ± 0.22* *0.79 ± 0.38*	*0.65 ± 0.19* *0.71 ± 0.28*	*0.52 ± 0.20* *0.65 ± 0.18*	0.221	< 0.001	1-*3< 0.001*	0.104
	Power absorbed in stance phase (W/Kg)	PD+NPS PD-NPS	-*0.63 ± 0.30* -*0.76 ± 0.39*	-*0.62 ± 0.33* -*0.67 ± 0.36*	-*0.51 ± 0.29* -*0.67 ± 0.26*	0.004	0.776	-	0.747
Knee	Extension moment in stance phase (N* m/Kg)	PD+NPS PD-NPS	*0.43 ± 0.22* *0.38 ± 0.16*	*0.45 ± 0.16* *0.41 ± 0.18*	*0.40 ± 0.19* *0.38 ± 0.16*	0.679	0.063	-	0.990
	Flexion moment in stance phase (N* m/Kg)	PD+NPS PD-NPS	-*0.31 ± 0.15* -*0.33 ± 0.17*	-*0.27 ± 0.13* -*0.39 ± 0.23*	-*0.28 ± 0.19* -*0.32± 0.19*	0.027	0.632	-	0.401
	Power generated in stance phase (W/Kg)	PD+NPS PD-NPS	*0.61 ± 0.21* *0.61 ± 0.39*	*0.66 ± 0.30* *0.55 ± 0.26*	*0.42 ± 0.17* *0.56 ± 0.19*	0.121	0.061	-	0.020
	Power absorbed in stance phase (W/Kg)	PD+NPS PD-NPS	-*0.83 ± 0.57* -*0.73 ± 0.38*	-*2.96 ± 6.91* -*6.40 ± 7.80*	-*7.59 ± 5.63* -*5.85 ± 7.07*	0.116	0.090	2-*3=0.035*	0.713
Ankle	Moment at loading response (N* m/Kg)	PD+NPS PD-NPS	*0.16 ± 0.08* *0.10 ± 0.08*	*0.15 ± 0.07* *0.17 ± 0.11*	*0.16 ± 0.08* *0.15 ± 0.10*	0.080	0.823	-	0.387
	Extension moment in stance phase (N* m/Kg)	PD+NPS PD-NPS	*1.22 ± 0.23* *1.28 ± 0.17*	*1.29 ± 0.30* *1.26 ± 0.29*	*1.15 ± 0.21* *1.28 ± 0.26*	0.846	0.265	-	0.079
	Power generated in stance phase (W/Kg)	PD+NPS PD-NPS	*2.41 ± 0.58* *2.22 ± 0.61*	*2.37 ± 0.53* *2.40 ± 0.63*	*1.61 ± 0.56* *1.86 ± 0.39*	0.489	< 0.001	1-*3< 0.001* *2-3< 0.001*	0.005
	Power absorbed in stance phase (W/Kg)	PD+NPS PD-NPS	-*0.34 ± 0.21* -*0.28 ± 0.17*	-*0.30 ± 0.26* -*0.32 ± 0.21*	-*0.24 ± 0.21* -*0.27 ± 0.18*	0.464	0.366	-	0.286

PD+NPS: Parkinson’s disease patients with neuropsychiatric symptoms; PD-NPS: Parkinson’s disease patients without neuropsychiatric symptoms GAIT: Single Task; mot: Motor dual-task; COG: Cognitive Dual-Task.

As regards the kinetic variables, the analyses showed that PD+NPS patients reported significant differences in the flexion moments of knee (p= 0.027) and hip (p= 0.020), and the power absorbed by the hip (p= 0.004). Regarding the influence of task, the significant variables were ankle generated power, hip generated power (all with p< 0.001) and extension moment of hip (p= 0.003). Finally, concerning the group-task interaction, the significant variables were again ankle generated power (p= 0.005) and knee-generated power (p= 0.020). The post-hoc results confirmed the significance reported by the influence of the task highlighting that the variables differed in the gait-cog (1-3) and mot-cog (2-3) tasks. These findings could indicate that PD+NPS tend to have altered joint movements and reduced motor control. It is worth noting that results of post-hoc analysis on all gait variables showed no significant differences in the comparison between single and motor-dual tasks, thus indicating a negligible contribution of the motor-dual task on changing gait pattern in our study population. This is the reason why, in the subsequent analyses, ML algorithms were implemented only on the variables of GAIT and COG dual task.

### C. ML Analysis

The classification analysis was initially performed using all gait features of GAIT and COG tasks, separately for spatio-temporal, kinematic, and kinetic parameters. However, only the evaluation metrics after the implementation of the Chi-Square feature selection are reported since they were more relevant. In particular, the most recurrent features selected by the feature selection algorithm referred to the single-task condition across all categories of gait variables. For each category of gait variables (spatio-temporal, kinematic, and kinetic), we report below the tables showing the metrics calculated during both the validation and testing phases.

**FIGURE 3.**
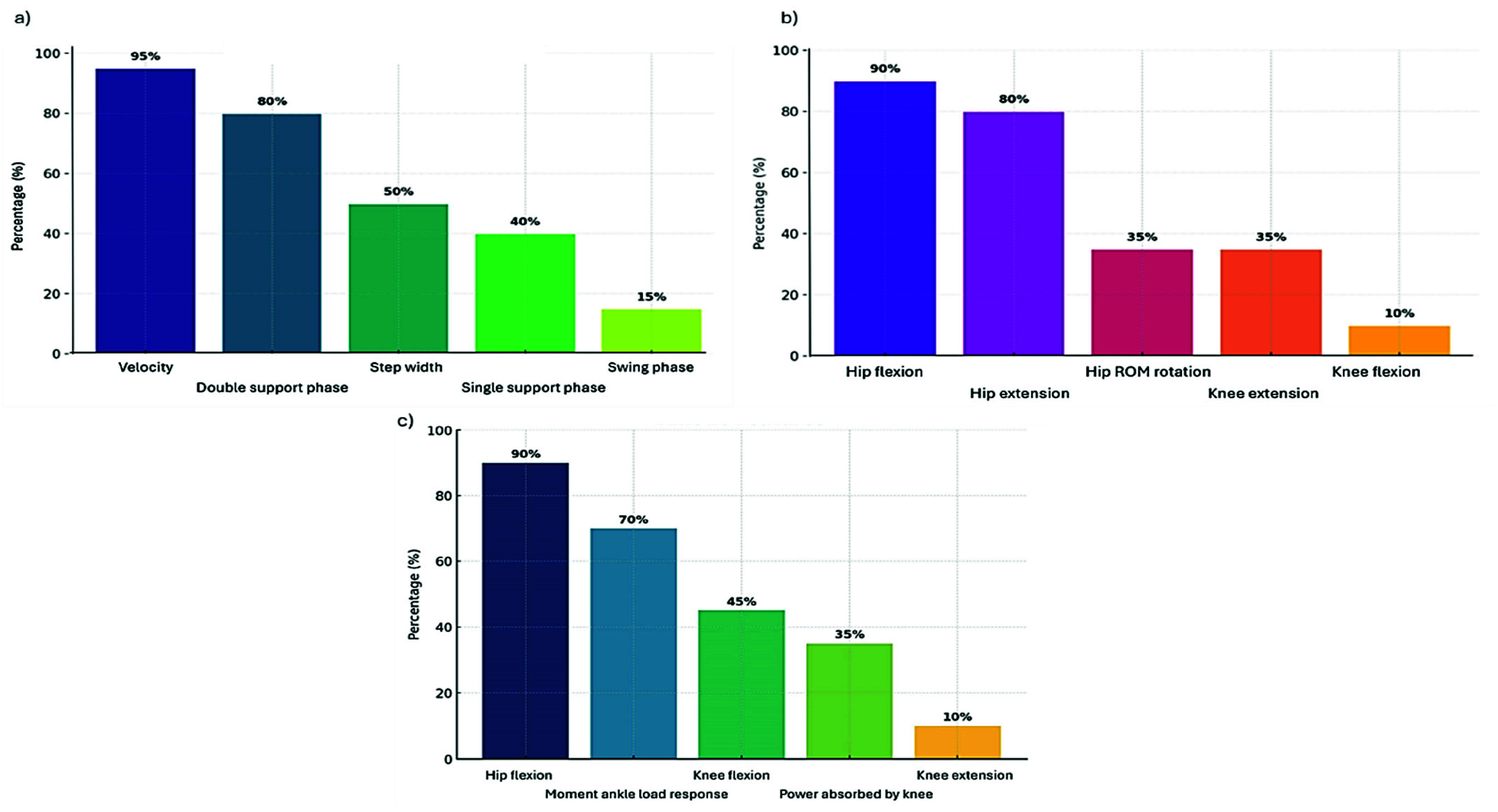
Bar plots showing the most influential gait features selected by the Chi-square algorithm across three domains: a) spatio-temporal parameter; b) kinematic parameters; c) kineticparameters.

Concerning the spatio-temporal variables, in the validation phase (Table 5) DT, KNN, and Ensemble achieved the highest accuracies (70.5%). In the testing phase, DT demonstrated the most stable performance (68.8% accuracy and the best balance between sensitivity (72.7%) and specificity (54.5%), making it the most reliable model. The extracted features used to train these models included double support phase, single support phase, step width, swing phase, and velocity. Among the spatio-temporal features, velocity (95%) and double support phase (80%) emerged as the most significant, underscoring their relevance in assessing overall gait stability and performance (Fig. 3a).

**TABLE 5:** Performance Values Evaluated in the Validation and Testing Phases of the Various Trained Classifier With Spatio-Temporal Variables

Classification Model Validation/Testing	Accuracy (%)		Sensitivity (%)		Specificity (%)		Precision (%)		AUCROC (%)	
DT	70.5	68.8	87.3	72.7	42.4	54.5	66.6	54.5	60.0	67.5
KNN	70.5	50.0	90.9	66.7	36.4	40.0	70.5	66.6	64.0	43.3
NB	62.5	43.8	85.5	66.7	24.2	30.0	50.0	60.0	58.8	55.0
SVM	68.5	43.8	90.9	100.0	30.3	10.0	66.6	100.0	63.1	48.3
DA	65.9	50.0	85.5	83.3	33.3	30.0	57.89	75.0	65.3	51.6
ENSEMBLE	70.5	50.0	87.3	100.0	42.4	20.0	66.6	100.0	65.8	50.0

Table 6 shows the performance metrics on the kinematic variables in validation and testing phase, respectively.

**TABLE 6:** Performance Values Evaluated in the Validation and Testing Phases of the Various Trained Classifier With Kinematic Variables

Classification Model Validation/Testing	Accuracy (%)		Sensitivity (%)		Specificity (%)		Precision (%)		AUCROC (%)	
DT	68.7	78.3	82.6	72.7	58.1	83.3	69.9	76.9	74.1	76.4
KNN	80.6	78.3	93.5	100.0	58.2	57.1	82.3	100.0	82.7	87.3
NB	74.6	82.7	83.1	100.0	58.1	66.4	68.0	100.0	71.6	88.1
SVM	74.6	91.3	79.5	100.0	66.8	83.3	85.0	100.0	73.1	83.1
DA	74.6	65.7	87.0	65.0	54.3	66.9	76.0	66.6	76.1	75.3
ENSEMBLE	76.6	82.7	87.0	81.8	61.3	83.3	76.0	83.3	81.5	87.3

In the validation phase, KNN achieved the highest accuracy (80.6%), followed by Ensemble (76.6%). In the testing phase, however, SVM proved to be the most reliable model, with an accuracy of 91.3% and an excellent balance between sensitivity (100%) and specificity (83.1%). The extracted features used to train the models were hip flexion, extension and ROM rotation, and knee flexion and extension.

Finally, as regards kinetic variables, in the validation phase (Table 7), ENSEMBLE showed the best performance (accuracy = 81.8%), followed by DT (80.3%) and SVM (78.9%). In the testing phase, however, NB proved to be the model with the highest accuracy (78.6%), but with limited specificity (50%); SVM maintained a better balance between sensitivity (62.5%) and specificity (66.7%) in testing, making it the most reliable model. The extracted features used for training the models included hip and knee flexion, the moment of ankle load response, and power absorbed by the knee. In the kinematic domain, hip flexion (90%) and hip extension (80%) emerged as the most influential joint ROM parameters, highlighting the critical role of proximal joint mobility in gait control (Fig. 3b). Similarly, the kinetic features emphasized the importance of hip flexion (90%) (Fig. 3c). These findings underscore the biomechanical significance of hip function across both kinematic and kinetic domains, reinforcing their central role in effective gait classification.

**TABLE 7:** Performance Values Evaluated in the Validation and Testing Phases of the Various Trained Classifier With Kinetic Variables

Classification Model Validation/Testing	Accuracy (%)		Sensitivity (%)		Specificity (%)		Precision (%)		AUCROC (%)	
DT	80.3	64.3	87.6	87.5	68.7	33.3	73.3	66.6	79.9	56.5
KNN	72.9	64.3	70.8	100.0	76.6	16.7	75.0	100.0	75.2	51.0
NB	77.9	78.6	79.6	100.0	75.7	50.0	63.1	100.0	78.2	71.5
SVM	78.9	64.3	80.4	62.5	76.7	66.7	71.8	57.1	75.8	59.8
DA	77.7	64.3	75.6	87.5	80.6	33.3	72.4	66.6	74.9	57.4
ENSEMBLE	81.8	64.3	89.1	75.0	68.7	50.0	81.4	60.0	88.7	64.8

## IV. Discussion

Conducted in a real-world outpatient clinical setting during routine visits, this study sought to explore gait alterations in PD patients as they relate to NPS. The novelty of the present study lies in its comprehensive gait assessment, which included the analysis of spatio-temporal, kinematic, and kinetic parameters, in a relevant cohort of PD patients (N= 104). The results highlighted gait analysis as a promising, non-invasive biomarker for identifying a PD phenotype with elevated NPS burden. Furthermore, the strong performance of ML models trained on gait data supports their potential as assistive tools to improve clinical decision-making, reduce subjectivity, and enable personalized patient monitoring. Notably, the relatively large sample size for a single-center Italian study enhances the robustness of the findings and highlights the potential for scalability and generalizability in future multicenter investigations.

Based on our results, this study demonstrates that gait analysis can be effectively integrated into clinical workflows for the management of patients with PD. This integration involves coordinated collaboration among neurologists, physiotherapists, and biomedical engineers, who together leverage objective and quantifiable gait metrics to obtain a comprehensive understanding of each patient’s motor function. In this way, clinicians can achieve more precise patient stratification, facilitate continuous monitoring, and better assess disease progression or therapeutic response, ultimately leading to a more personalized, proactive, and effective model of care.

In detail, when assessing demographic and clinical variables, the two groups resulted comparable matched, apart from H&Y score, MDS-UPDRS I and II sub-scores and total score, and for MoCA test. The difference on MDS-UPDRS since the classification of the two groups of the present study was based on such a score. As regards H&Y score and MDS-UPDRS II, with the first scale assessing the stage of PD and the second score mirroring patients’ performance on daily living activities, our findings prove largely consistent with previous literature [35], [36], [37], [38]. Finally, MDS-UPDRS total score is clearly driven by the rating of the first two parts of the scale.

The two-way ANOVA results for spatio-temporal variables indicated that PD+NPS patients compared with those without exhibited impaired gait, demonstrating slower velocity and greater instability. Kinematic analysis revealed that patients with NPS exhibited altered joint motion patterns. These included increased flexion and decreased extension at hip, knee and ankle with concomitant ROM limitation leading to a shuffling gait pattern. Kinetic analysis showed that patients with NPS exhibited dysfunctional motor control at the joint level, mainly due to a reduced capacity for force generation.

Collectively, spatio-temporal, kinematic and kinetic findings indicate that PD+NPS patients showed more dynamic instability, increased postural dysfunction and altered forces distribution on lower limbs. ML analyses corroborated these results, demonstrating the importance of kinematic and kinetic variables for classifying PD patients based on their mental status. Importantly, these quantitative gait features are highly interpretable from a clinical perspective, as they map directly onto observable motor impairments and well-known compensatory strategies in PD. This enhances the explainability of the extracted features, facilitating not only their integration into ML models but also their translation into meaningful clinical insights for patient stratification and personalized management.

It is worth noting that the study is based on real-world clinical data and includes an external validation on an independent dataset derived from real clinical practice, thereby confirming the robustness and generalizability of the ML models beyond the development cohort. Interestingly, the classification of patients was more influenced by the single task rather than the dual-task conditions. Conversely, in the study by Russo et al. [15], the classification was influenced by the cognitive dual task in the comparison between PD patients with and without mild cognitive impairment. Such difference may be attributed to the fact that, in the present study, both groups were similar on objective cognitive measures as indicated by comparable scores on the MoCA test, a common screening tool for cognitive impairment in PD. As a result, the cognitive dual-task condition may not have provided additional discriminative power compared to the single-task assessment.

The classification models were trained on the most recurrent gait variables selected by the Chi-square selection algorithm. DT, KNN, and Ensemble models showed promising performance with spatio-temporal variables, with DT emerging as the most stable performer in the testing phase. However, kinematic variables were better classified using SVM, which achieved the highest testing performance, especially in terms of sensitivity. Kinetic variables showed strong performance with the ensemble model during the validation phase, but SVM maintained better reliability in the testing phase, demonstrating a good balance of sensitivity and specificity.

These results reinforce the clear association between gait impairment and the presence of NPS in PD patients, particularly emphasizing increased instability and slowness [15]. Integrating gait analysis into PD monitoring enables a more proactive and personalized approach to managing the motor and non-motor features of the disease. This method does not replace traditional clinical standards but complements them by providing objective and quantitative data that support early detection of gait abnormalities, allowing timely interventions and targeted therapies, especially in the early stages or in patients with multiple comorbidities. This highlights the clinical relevance of gait analysis, not only as a diagnostic and monitoring tool, but also as a valuable outcome measure in clinical trials.

Additionally the present findings further expand the relationship between motor and non-motor symptoms in PD highlighting that the connection between gait and mental symptoms extends beyond cognitive status [15], [39], [40]. Movement analysis protocols based on both single- and dual-task paradigms, which allow for the extraction of kinematic and kinetic parameters, offer a scalable and adaptable approach across various neurodegenerative diseases. These quantitative biomarkers can subsequently be leveraged in ML models, supporting automated classification, progression tracking, and personalized intervention strategies. As already demonstrated by the present research group, this approach shows strong translational potential and can be effectively integrated into both clinical and research settings [15], [29]. Due to the limited published data on the kinematic and kinetic characteristics in PD patients with distinctive non-motor symptoms, many of our findings remain speculative and require further confirmation. Future developments should aim to extend this work into a multicenter framework, allowing for the validation of findings across different clinical contexts and patient populations. Additional studies are needed to verify and expand upon these results, for evaluating the robustness of the extracted features and validating model performance on new datasets and longitudinal follow-up data. This will be essential to assess both the stability of the models over time and their potential impact on clinical workflows and patient outcomes in real-world settings.

Despite the promising results of this study, some methodological and practical aspects should be considered. First, the methodology relies on high-quality motion analysis equipment, which may not be readily available in all clinical settings due to costs and logistical requirements. In addition, the physical space needed for equipment installation and the technical expertise required for data acquisition and processing may limit broader implementation in routine healthcare practice. Furthermore, movement analysis requires approximately 30 minutes of acquisition time, a duration that can still be considered feasible within an outpatient clinical setting. However, because recordings needed to be performed strictly during the patients’ optimal ON phase, evaluations had to be scheduled within specific time windows to ensure appropriate alignment with medication intake.

Nevertheless, the computational requirements for data processing and model application are relatively low. In fact, the collected data can be processed efficiently using standard laptops, making the workflow technically feasible for integration into routine clinical practice once the acquisition infrastructure is available.

Finally, this study did not include a healthy control group, which could have provided a clearer distinction between gait abnormalities associated with PD itself and those specifically related to the presence of additional neuropsychiatric symptoms. Future studies should therefore include healthy controls in order to better characterize the specific contribution of mental status alterations to gait impairment in PD and may also explore wearable-based approaches to facilitate broader clinical application.

## V. Conclusion

The evidence that self-reported NPS are associated with distinct gait patterns further supports the concept that specific disease phenotypes exist, in which motor and non-motor symptoms jointly contribute to the overall clinical presentation. Recognizing that both domains contribute to disease presentation may promote the implementation of integrated tools of phenotyping, also in order to enable earlier therapeutic interventions that do not neglect either component of the disease.

## Conflicts of Interest

The authors have no relevant conflicts of interest to disclose.

## Author Contributions

Conceptualization: Carlo Ricciardi, Marianna Amboni, and Maria Romano; Data curation: Federico di Filippo, Giuseppe de Biasi, and Paolo Barone; Formal analysis and Visualization: Maria Romano, Carlo Ricciardi, Martina Mestizia, Noemi Pisani, and Alfonso M. Ponsiglione; Investigation: Federico di Filippo, Giuseppe de Biasi, Paolo Barone, and Marianna Amboni; Methodology: Maria Romano, Carlo Ricciardi, Alfonso M. Ponsiglione, and Maria Romano; Software: Maria Romano, Carlo Ricciardi, Martina Mestizia, and Noemi Pisani; Supervision: Marianna Amboni and Maria Romano; Validation: Federico di Filippo, Giuseppe de Biasi, Paolo Barone, and Marianna Amboni; Writing—original draft: Maria Romano, Carlo Ricciardi, Martina Mestizia, Federico di Filippo, and Noemi Pisani; Writin—review and editing: Carlo Ricciardi, Alfonso M. Ponsiglione, Paolo Barone, Marianna Amboni, and Maria Romano.

## Ethical Considerations

The informed permission was provided by the patients, and the study was authorized by the Local Ethical Committee Campania Sud with registry number “P.U. n.2_rpsc/2020” on 02/12/2020. Furthermore, data was anonymized before the analysis was conducted.

## Data Availability

The dataset presented in this article is not readily available because they cannot be made publicly accessible, as the informed consent signed by the participants prohibits data sharing on an individual level.
